# Autophagy-related proteins measured in umbilical blood cord samples from human newborns: what can we learn from?

**DOI:** 10.1038/s41390-024-03382-2

**Published:** 2024-07-16

**Authors:** Vanessa Ginet, Julien Puyal, Anita C. Truttmann

**Affiliations:** 1 Department of Women, Mother and Child, Clinic of Neonatology, University Hospital Center of Vaud, Lausanne, Switzerland; 2 Department of Fundamental Neurosciences, University of Lausanne, Lausanne, Switzerland; 3 CURML, University Center of Legal Medicine, Lausanne University Hospital, Lausanne, Switzerland

Comment to Künstle, N. et al. Differences in autophagy marker levels at birth in preterm vs. term infants. *Pediatr Res* (2024). 10.1038/s41390-024-03273-6.

**Fig. 1:**
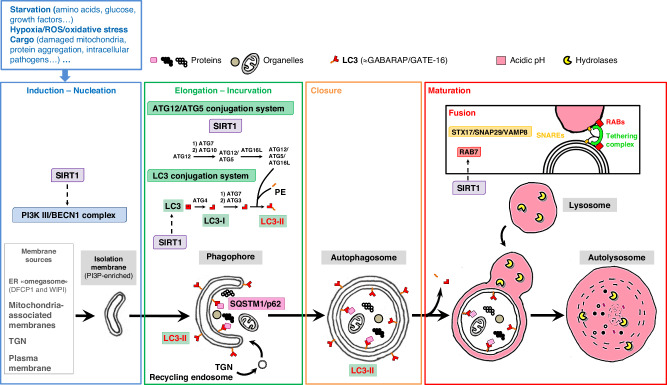
Overview of mammalian autophagy. Autophagy involves the sequestration of cargos (such as long-lived proteins, organelles, and pathogens) by an intermediate multi-membrane organelle called the autophagosome, which then fuses with a lysosome to form an autolysosome for degradation. Autophagy can be divided into 4 main steps: induction/nucleation, elongation/incurvation, closure and maturation. Autophagy is induced by different cell stresses (such as starvation, oxidative stress, accumulation of defective proteins or unfunctional organelles) and depends on several protein complexes (not exhaustively represented in this figure). The PI3K-III (class III phosphoinositide 3-kinase)/BECN1 (BECLIN 1) complex drives the nucleation of the isolation membrane. Autophagosome membrane components could have different sources such as ER omegasome, mitochondria-associated membrane, trans-Golgi network (TGN) or plasma membrane. PI3K-III activation and generation of phosphatidylinositol 3-phosphate (PI3P) on isolation membrane serves as a signal for protein assembly required for autophagosome formation. LC3-II is a key autophagic protein playing important roles in elongation, cargo selection, incurvation, and closure steps of autophagy. A LC3 cytosolic precursor is first post-translationally modified (LC3-I) and then covalently conjugated to the membrane phospholipid phosphatidylethanolamine (PE) (LC3-II). This lipidated form is recruited at the phagophore membrane and remains on autophagosome membrane until maturation into a degradative autolysosome (acidification and loading in hydrolases). Fusion machinery involves RAB proteins (mainly RAB7) which recruit tethering complexes (HOPS, TECPR1) to bring the two vesicular compartments close enough to allow SNARE proteins to drive the fusion. SIRT1 is a NAD^+^-dependent histone deacetylase involved in various processes, including cell proliferation, survival, differentiation, oxidative stress, and autophagy. SIRT1 has been shown to regulate autophagy at different steps (induction, elongation and fusion). SQSTM1/p62 is an important autophagy receptor recruiting many cargoes (ubiquitinated substrates, mitochondria, bacteria…) into autophagosomes, and selectively degraded by autophagy.

Macroautophagy (hereafter simply referred to as autophagy) is a highly conserved intracellular process involved in the degradation and recycling of proteins and organelles.^1^ It cooperates with the ubiquitin-proteasome system to maintain cell homeostasis and stress adaptation. Defective autophagy results in the buildup of dysfunctional proteins and organelles, abnormal protein aggregates, or invading pathogens, and thus contributes to various pathologies such as aging, cancer, proteinopathies (e.g. Alzheimer’s and Parkinson’s diseases), or infection. In most cases, stress-enhanced autophagy flux promotes cell survival, for instance during starvation or pathogen infection. However, after reaching a certain threshold or in specific circumstances, exacerbated autophagic mechanisms can contribute to cell death (e.g. in neurons after perinatal cerebral hypoxia-ischemia^2–4^). In short, maintaining an appropriate level of autophagic flux, from the engulfment of cargoes into an autophagosome to their fusion with a lysosome (maturation) for degradation (Fig. 1), is crucial for cell well-being and survival. Most autophagy-deficient mice typically do not survive beyond the embryonic or neonatal stage.^5^ From the first mention of the term “autophagy” over sixty years ago, research on autophagy continues to progress and gain importance in numerous fields such as cancerology, neurosciences, and immunology. Autophagy also plays a significant role in various physiological and pathological processes in mammalian development, including embryogenesis, morphogenesis, maternal birth induction, and postnatal organ adaptation.^6–10^

Among the best-investigated cellular stressors that enhance autophagy, reactive oxygen species (ROS) can be considered as a key mechanism.^11,12^ ROS (or oxidative stress) results from the imbalance between ROS production and the available antioxidative defenses. Preterm infants are known to be prone to oxidative stress, particularly due to immature or deficient antioxidative enzymes.^13^ Therefore, when born prematurely, the infants are exposed to several exogenic and endogenic stressors during their hospitalization (infections, nutrition, drugs, infections, ventilation-induced lung injury, sensorial stress) and this may ultimately lead to inflammation-driven pathologies, such as preterm brain injury or bronchopulmonary dysplasia. In general, the lower the gestational age and the sicker the infant, the more pronounced are the implications on the different organs.^13^ Further, oxidative stress has been recently associated with several adverse pregnancy outcomes, such as pre-eclampsia, fetal growth restriction, gestational diabetes mellitus, and preterm birth.^12^ The main organ involved in these pathological processes is the placental tissue, mainly the placentation which is considered as a transient vital fetal organ impacting maternal and fetal outcomes.^14^ According primarily to data from preclinical models, an impaired placental autophagy flux appeared to be one of the possible cellular mechanisms contributing to pregnancy-associated pathologies, specifically to preterm birth.^14^ Restricted growth of the placenta leading to hypertension in the dam is also observed when autophagy is specifically deficient in trophoblasts.^15^ Moreover, efficient autophagy flux is necessary to support placental inflammation resistance and prevent inflammation-induced preterm birth.^14,16–18^ Limited information on the levels of autophagy flux over human gestation is available, and the few existing data are restricted to the placental tissue. Cao et al. suggested that preterm placentas present less active autophagy than term infants as shown by a lower expression of LC3-II (a marker of autophagosome (Fig. 1)) combined with a higher expression of SQSTM1/p62 (an autophagy receptor selectively degraded by autophagy) in preterm samples.^17^ Other studies have reported higher SQSTM1/p62 expression in preterm placentas, but data for LC3-II levels are more heterogeneous.^19,20^

Based on these previous findings, Künstle et al. hypothesized in the recent *Pediatric Research* issue of May 29 that preterm infants may begin life with different levels of autophagy from those of term infants. They performed an exploratory study that presents, for the first time, biological human data of autophagy-related protein levels in umbilical cord blood plasma of both preterm and term infants in a large population-based sample.^21^ The population studied is issued from the BILD (Bern-Basel Infant Lung Development) prospective cohort study (1999–2019) including more than 500 infants (453 term and 64 preterm infants less than 37 weeks of gestation divided into 4 groups). This cohort has been studied previously for developmental and environmental aspects, focusing on the lung development and respiratory morbidity of preterm and term infants.^22^ Comparisons stratified on gestational age were performed in adjusted models for different variables (such as preeclampsia, fetal growth restriction, gestational diabetes, antibiotic use in mothers and chorioamnionitis). Among the investigated proteins (LC3B, SQSTM1/p62, BECLIN1 (BECN1), and SIRT1), no statistical differences were obtained for BECN1 and LC3B, two ATG proteins involved in autophagy induction and autophagosome formation, respectively.^1^ However, SQSTM1/p62 levels were significantly reduced (up to 38th week) with gestational age and negatively correlated with LC3B levels, while SIRT1 (an indirect non-specific autophagy activator) levels were increased. Altogether, these data suggest that autophagy activity could be, just after birth, lower in preterm than term infants. Künstle et al. proposed that differences in autophagy-related proteins may be linked to different responses and vulnerabilities according to their outcomes, specifically respiratory morbidity.

Despite these interesting findings, experimental limitations in human samples require caution in concluding on the levels of the autophagy flux and the roles of autophagy. An increased autophagic flux means that all autophagy steps are enhanced, from the formation of autophagosomes to their maturation (Fig. 1). A decreased or defective autophagy could result from either a reduced induction/formation of autophagosomes or a failure in autophagosome maturation (or both). Increased LC3-II levels can thus reflect enhanced autophagy flux (more newly formed autophagosomes) or impaired degradation (autophagosome accumulation). To be conclusive, studies on autophagy flux should investigate at least LC3-II and SQSTM1/p62 levels. Künstle et al. showed no significant difference in LC3B levels. However, the method used to evaluate protein level did not allow to differentiate between the cytosolic LC3-I form, and the autophagosome-bound LC3-II form. Therefore, it is difficult to conclude on autophagosome presence in the blood cord over gestation. The higher SQSTM1/p62 expression observed in preterm could suggest that autophagic degradation of this protein is lower in preterm than in term infants. Lower SIRT1 expression in preterm tends to confirm a potentially less active autophagy flux in preterm. However, with no indication of autophagosome abundance, a developmental change in SQSTM1/p62 expression level between preterm and term could not be excluded.

Moreover, autophagic flux is an adaptative process that changes over time and is modulated by external changes. Therefore, one could speculate that the findings reported are time- and maturation-dependent and might be associated with several pathological conditions (gestational diabetes, chorioamnionitis, pre-eclampsia). The authors have considered this by adjusting their analysis for risk factors. Serial measurements of autophagic markers in blood, throughout hospitalization could be one potential approach to reveal more information about this issue.

It is also important to note that the functional significance and the origin of the autophagic markers measured in cord blood plasma just after birth are undefined and could be diverse. As the authors discussed, exocytosis and extracellular vesicles release in plasma could be one explanation for their findings. Recently, secretory autophagy has also been recognized as an unconventional pathway for extracellular secretion.^23^ Then, the plasma level of autophagy-related proteins does not necessarily reflect the intracellular level of autophagy. However, regardless of the origin of these proteins, the results of this issue indicate that it would be very interesting to determine whether the levels of autophagy-related proteins at birth could be used as predictive biomarkers for subsequent complications. It would permit to categorize and individualize the management of these identified patients. For instance, in adult neurology, SQSTM1/p62 blood levels could be used as a biomarker to evaluate the severity of Charcot-Marie-Tooth disease type 1A.^24^

In conclusion, this study provides new descriptive insights into a potential cellular mechanism that may be related not only to preterm birth, but also to the greater susceptibility of preterm infants later in life. Further research is needed to clarify the significance of these gestational changes in autophagy-related proteins in blood cord plasma. To gain further insight into the correlation between autophagy and preterm complications, similar investigations could be conducted on samples obtained from other postnatal biological fluids, including but not limited to tracheal secretions, plasma, urine, cerebrospinal fluids, and mucous and skin smears.
